# Long-term follow-up of patients with elevated serum calcium concentrations in Swedish primary care

**DOI:** 10.3109/02813432.2013.861152

**Published:** 2013-12

**Authors:** Sofia Dalemo, Robert Eggertsen, Per Hjerpe, Svante Jansson, Erik G. Almqvist, Kristina Bengtsson Boström

**Affiliations:** ^1^Institute of Medicine, Department of Public Health and Community, Primary Health Care, Sahlgrenska Academy, University of Gothenburg, Gothenburg, Sweden; ^2^R&D Centre Skaraborg Primary Care, Skövde, Sweden; ^3^Mölnlycke Primary Health Care and Research Centre, Sweden; ^4^Institute of Clinical Sciences/Department of Surgery, Sahlgrenska Academy, University of Gothenburg, Gothenburg, Sweden; ^5^Department of Internal Medicine, Skaraborg Hospital, Skövde, Sweden

**Keywords:** Gender, general practice, hypercalcaemia, mortality, longitudinal studies, primary care, primary hyperparathyroidism, Sweden

## Abstract

**Objective:**

To follow up patients with elevated calcium concentrations after 10 years.

**Design:**

Longitudinal, using medical records, questionnaires, and clinical investigation.

**Setting:**

Primary care in Tibro, Sweden, 2008–2010.

**Subjects:**

127 patents with elevated calcium concentrations and 254 patients with normal calcium concentrations from the local community, attending the health care centre.

**Main outcome measures:**

Diagnoses and mortality in patients with elevated calcium concentrations in 1995–2000, compared with patients with normal calcium concentrations and the background population.

**Results:**

The proportion of patients for whom no underlying cause was detected decreased from 55% at baseline to 12% at follow-up. Primary hyperparathyroidism was most common in women, 23% at baseline and 36% at follow-up, and the cancer prevalence increased from 5% to 12% in patients with elevated calcium concentration. Mortality tended to be higher in men with elevated calcium concentrations compared with men with normal calcium concentrations, and was significantly higher than in the background population (SMR 2.3, 95% CI 1.3–3.8). Cancer mortality was significantly increased in men (p = 0.039). Low calcium concentrations were also associated with higher mortality (p = 0.004), compared with patients with normal calcium concentrations.

**Conclusion:**

This study underscores the importance of investigating patients with increased calcium concentrations suggesting that most of these patients – 88% in our study – will turn out to have an underlying disease associated with hypercalcaemia during a 10-year follow-up period. Elevated calcium concentrations had a different disease pattern in men and women, with men showing increased cancer mortality in this study.

A possible underlying cause of elevated calcium concentrations was found at baseline in 45% of 127 primary care patients.

- At the 10-year follow-up, there was an association with a condition potentially related to hypercalcaemia in 88% of the patients.
- Elevated calcium concentrations were mainly associated with primary hyperparathyroidism in female patients and with malignancies and increased mortality in male patients, compared with controls.
- It seems important to recheck elevated calcium concentrations as the levels may fluctuate.

## Introduction

Several diseases can cause elevated calcium concentrations in serum and plasma, the most common being primary hyperparathyroidism (pHPT) and malignant diseases. The calcium concentration has been a routine laboratory analysis for decades and is used to screen for diseases, especially for pHPT, which is difficult to detect as the symptoms are often vague [1].

In the 1990s, some health care centres (HCCs) in Sweden were equipped with point-of-care tests to analyse calcium, which was encouraged by the authorities, but the number of tests performed varied considerably between physicians [2–4].

Hospitalized patients often have more pronounced hypercalcaemia with aggravated symptoms, compared with patients in primary care; hence their diagnoses are often more obvious. In a previous study we showed that no underlying cause was found in 55% of the patients with elevated calcium concentrations in primary care [3]. As the calcium concentration is often included in primary care routine analyses, more knowledge is needed concerning the effects of increased calcium concentrations on morbidity and mortality. As very few studies have been performed on hypercalcaemia in primary care, there is a need for long-term studies of patients with elevated calcium concentrations [5].

The primary objective of this study was to investigate the long-term mortality and morbidity in patients with elevated calcium levels, analysed between 1995 and 2000, and in whom no immediate underlying diagnosis was found. The secondary objective was to study mortality in this cohort compared with normocalcaemic patients.

## Material and methods

Tibro is a rural community with 11 000 inhabitants and one HCC. The medical records of all patients with elevated calcium concentrations between 1995 and 2000 have previously been studied [3]. Between 1993 and 2006, calcium was analysed in whole blood at the HCC laboratory in Tibro by Vision, Abbott. At baseline, 1995–2000, an elevated calcium concentration was defined as at least one calcium test ≥ 2.56 mmol/l. Two age and sex-matched controls with calcium < 2.45 mmol/l were selected for each patient. Age was matched within two months, but for the oldest (n = 2), the match was within three years. Patients with calcium concentrations between 2.45 and 2.55 mmol/L were excluded from the control group.

### Study subjects

**Figure 1.**
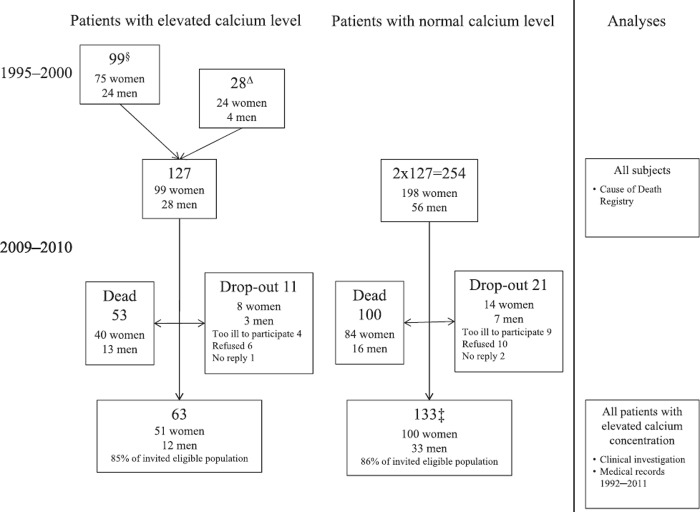
Flow chart of patients with elevated calcium concentrations at Tibro Health Care Centre, Sweden, 1995–2000, and re-examination of the patients with elevated and normal calcium concentrations during 2009–2010. Notes: ^§^Included in our previous study [3]. ^∆^Patients with hypercalcaemia at baseline not included in previous study because of technical problems. ^‡^ Two persons only answered the questionnaire, no laboratory samples.

A flow chart of study subjects is shown in Figure 1. Study subjects were invited by mail to participate in the follow-up to July 2011. Non-responders were contacted by telephone. Subjects who had moved were interviewed by phone and blood samples were taken at their local HCC.

For patients with elevated calcium concentrations, results from laboratory and radiological examinations and consultant opinions, as detailed in their medical records for the years 1992–2011 (ProfDoc Journal III, ProfDoc AB), were studied to find an underlying diagnosis associated with hypercalcaemia. Further information, updated on 1 August 2011, was derived from the National Swedish Cause of Death Register, for patients with both elevated and normal calcium concentrations. Data on mortality in the background population in Tibro community from 1995 to 2010 were collected from the National Swedish Cause of Death Register.

### Clinical investigation

A nurse interviewed all the participants regarding their former and current diseases. Self-reported alcohol consumption and current medication were recorded. Weight and height were measured and non-fasting blood samples were obtained. Complete blood count and sedimentation rate in whole blood were analysed locally by Sysmex KX 21 (Sysmex) and by Micro sed system (Elekta lab). Calcium, albumin, creatinine, and alkaline phosphates in plasma were analysed centrally in the primary care laboratory by Integra 400+ (Roche). There was no difference in the reference intervals for analyses of calcium in plasma and serum [6]. Serum intact parathyroid hormone was analysed by two antibodies and ionized calcium by ion-selective electrode by Unilabs, Skövde, using Centaur XP (Siemens) and ABL 800 (Radiometer).

A laboratory assistant at the health care centre laboratory performed the laboratory analyses. Internal quality controls were performed regularly. All the laboratories of the primary health care centres in the area were certified by the Swedish Board for Accreditation and Conformity Assessment (SWEDAC), assuring adherence to the ISO 15 189 standard, in September 1998. A quality assessment of the calcium analyses in Tibro during 1995–2000 was made by retrieving data from national external controls, performed by Equalis, Uppsala [7]. Quality controls revealed that between January 1997 and April 1998, when parts of the analyses for this study were performed, the calcium analysis deviated above defined quality levels in several instances (data not shown). Forty-five patients with high calcium concentrations only had a single, initial high calcium level, but normal calcium concentrations at follow-up. Most of these samples were taken between January 1997 and April 1998. We found no underlying diagnoses at follow-up in any of these 45 patients. They were therefore excluded from the analyses.

### Statistical analyses

Descriptive statistics were presented and comparisons were performed using the t-test. The mortality was analysed with the chi-squared test and Crosstabs. A comparison between the groups of survival time was performed with a Kaplan–Meier survival analysis with log-rank test. The mortality among men and women with elevated calcium concentrations was also compared with the mortality among inhabitants in Tibro, using standardized (with respect to age group and time period) mortality ratios (SMR), and was expressed as an odds ratio (OR) presented with a 95% confidence interval (CI). For the mortality among inhabitants in Tibro the data from the Swedish Death Register were used and for the comparisons and the calculations of person-years at risk and SMR were performed with software PAMCOM [8–11]. To analyse the impact of different levels of calcium on mortality, patients were divided into three groups according to their calcium levels (≤ 2.30, 2.31–2.46, ≥ 2.56 mmol/L). A p-value of < 0.05 was considered statistically significant. All statistical analyses except the SMR were performed using the SPSS 20 statistical package.

## Results

During the follow-up period, 153 of the 381 participants died and 32 dropped out for other reasons (see Figure 1). Thus, the number of subjects examined was 196, which was 86% of the eligible population. For all 381 participants the median follow-up time was 10.1 years (range 0.0 to 16.6 years), resulting in 3863 person years. The characteristics of the study subjects are presented in Table I. There was no difference in the number of smokers. Patients with elevated calcium concentrations had a higher mean corpuscular volume. This could not be explained by differences in alcohol consumption, as the patients with elevated calcium concentrations had significantly lower alcohol consumption. At follow-up, patients with an elevated calcium concentration had a higher mean age than the normocalcaemic patients; however, the difference was not significant.

**Table I.** Characteristics of patients with elevated and normal calcium concentrations, 1995–2000 and 2008–2010, men and women, at Tibro Health Care Centre, Sweden.

	Patients with elevated calcium concentration						Patients with normal calcium concentration						Comparison between patients with elevated and normal calcium concentrations		
Variables	Total Mean	Percentile	Men Mean	Percentile	Women Mean	Percentile	Total Mean	Percentile	Men Mean	Percentile	Women Mean	Percentile	Total p-value	Men p-value	Women p-value
1995–2000															
Number	127		28		99		254		56		198				
Age, 1 January 1995	61.4	36.6, 80.0	55.6	31.8, 77.0	63.0	38.8, 80.2	61.3	36.3, 80.0	55.5	39.0, 80.3	62.9	31.0, 77.0	n.s.	n.s.	n.s.
Age range	18–94		18–82		18–94		18–91		18–82		18–91				
S-Calcium^1^	2.66	2.56, 2.78	2.72	2.57, 2.88	2.64	2.56, 2.78	2.33	2.21, 2.43	2.31	2.19, 2.42	2.33	2.21, 2.44	< 0.001	< 0.001	< 0.001
2008–2010															
Number	63		12		51		133		33		100				
Age, 1 January 2011	71.4	49.4, 89.0	67.7	49.2, 80.0	72.3	52.0, 89.0	70.0	45.2, 88.0	66.8	45.2, 84.4	71.0	45.8, 90.0	n.s.	n.s.	n.s.
Age range	31–97		45–82		31–97		30–99		31–99		30–97				
P-calcium^2^	2.42	2.26, 2.59	2.39	2.18, 2.55	2.43	2.27, 2.64	2.35	2.22, 2.47	2.32	2.21, 2.42	2.36	2.23, 2.48	< 0.001	0.085	0.003
Ionised calcium^3^	1.29	1.22, 1.40	1.28	1.24, 1.31	1.29	1.22, 1.44	1.23	1.18, 1.27	1.22	1.16, 1.26	1.23	1.18, 1.28	< 0.001	< 0.001	< 0.001
Creatinine^4^	79	57, 122	96	72, 139	75	56, 109	74	52, 98	91	68, 125	69	50, 92	n.s.	n.s.	0.037
Parathyroid hormone^5^	68	20, 118	60	14, 136	69	21, 113	58	26, 107	57	22, 104	59	28, 113	n.s.	n.s.	n.s.
Haemoglobin^6^	133	114, 149	143	122, 165	131	111, 146	131	116, 148	136	116, 154	129	116, 146	n.s.	n.s.	n.s.
ESR^7^	17	3, 49	12	2, 46	18	3, 53	15	3, 33	14	2, 47	15	5, 33	n.s.	n.s.	n.s.
MCV^8^	92	86, 99	92	86, 100	92	86, 99	90	85, 95	90	84, 96	90	85, 95	< 0.001	n.s.	0.001
ALP^9^	1.2	0.8, 1.6	1.2	0.7, 1.7	1.2	0.8, 1.6	1.2	0.8, 1.7	1.2	0.8, 1.5	1.3	0.8, 1.8	n.s.	n.s.	n.s.
Albumin^10^	43	39, 48	44	38, 49	43	40, 48	43	39, 46	43	38, 47	43	40, 46	n.s.	n.s.	n.s.

Notes: Percentile, 10th and 90th percentile. Data are means (SD), n.s. = not significant, Age in years = y. M = man, W = woman, Reference range.

^1^Serum calcium analysed in whole blood (mmol/L) 2.15–2.55.

^2^Plasma calcium (mmol/L)2.15–2.50.

^3^Ionised calcium (mmol/L) 1.15–1.35.

^4^Creatinine (μmol/L) M 60–100, W 50–90.

^5^Parathyroid hormone (ng/L) 15–65.

^6^Haemoglobin (g/L) M 122–166, W 113–153.

^7^Erythrocyte sedimentation rate (mm) M < 20, W < 30.

^8^MCV = mean corpuscular volume (fL) 82–102.

^9^ALP = alkaline phosphatase (μkat/l) 0.6–1.8.

^10^Albumin (g/L) 18–40y 36–48, 41–70 y 36–45, > 70 y 34–45.

Additional diagnoses compared with baseline in patients with elevated calcium concentrations are given in Table II. This table comprises all patients with elevated calcium concentrations including deceased patients, dropouts, and participants. Many diagnoses increased between the two observations, the most common being pHPT. Some 43% of the patients with pHPT had undergone surgery. In 12% of the patients the cause of the elevated calcium concentration remained unknown. The majority of these individuals had a single, unconfirmed, increased calcium level.

**Table II.** Comparison between diagnoses in percentage of patients with elevated calcium concentrations at first examination in 1995–2000 at Tibro Health Care Centre and diagnoses found after second clinical examination and register investigation in 2009–2010.

	First investigation				Second investigation			
	Number	%	Men	Women	Number	%	Men	Women
Primary hyper-parathyroidism	29	23	3	26	46	36	6	40
Parathyroidectomy	11		2	9	20		2	18
Cancer	6^1^	5	3	3	15^2^	12	7	8
Vitamin D medication	5	4	0	5	15	12	1	14
Sarcoidosis	1	1	1	0	1	1	1	0
Kidney disease	5	4	2	3	14	11	5	9
Lithium treatment	1	1	0	1	2	2	0	2
Transient thyroiditis	2	1	0	2	5	4	0	5
Skeletal disease	8^3^	6	3	5	13^4^	10	4	9
Unresolved elevated calcium	70	55	16	54	16^5^	12	4	12
Total	127	100	28	99	127	100	28	99

Notes: Numbers in italics represent subgroups of diagnoses:

^1^1 prostate cancer, 1 breast cancer, 1 small cell lung cancer, 1 myeloma, 1 tonsil cancer, 1 anaplastic thyroid cancer.

^2^3 prostate cancer, 3 breast cancer, 1 small cell lung cancer, 1 myeloma, 1 tonsil cancer, 1 anaplastic thyroid cancer, 1 hepatic cancer, 1 malignant melanoma, 1 colorectal cancer, 1 pancreatic cancer, 1 ovarian cancer.

^3^2 osteomalacia, 4 fractures of a vertebra, 1 rib fracture, unfastened hip prosthesis.

^4^2 osteomalacia, 7 fractures of a vertebra, 1 osteitis, 1 rib fracture, 1 unfastened hip prosthesis, 1 RA = rheumatoid arthritis.

^5^12 were dead before the reinvestigation, 2 refused to participate, 2 participated.

**Figure 2.**
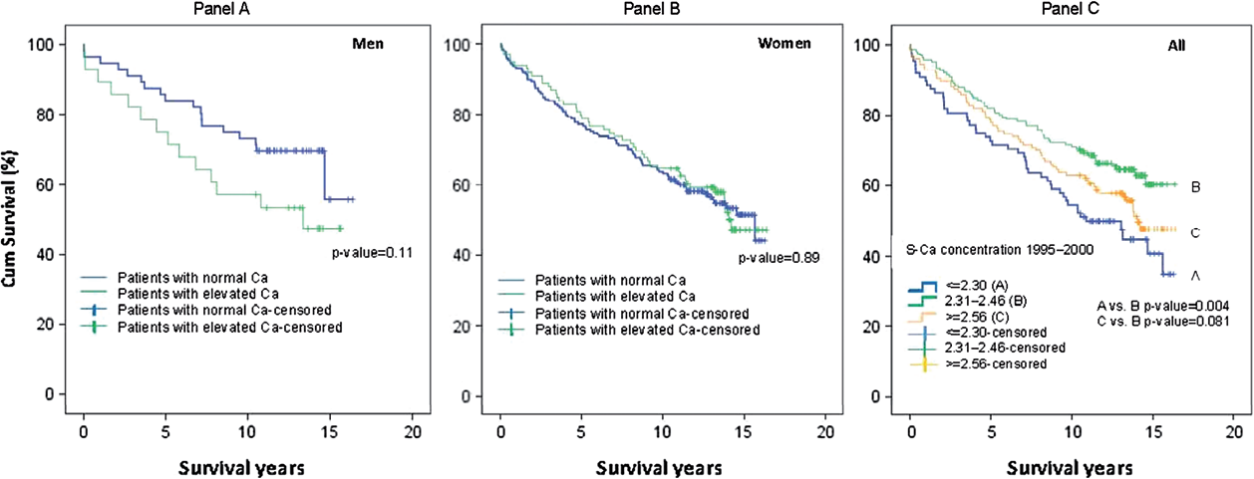
Mortality of patients with elevated (≥ 2.56 mmol/L) and normal (≤ 2.46 mmol/L) calcium concentrations, Panel A: men, Panel B: women, at Tibro Health Care Centre in 1995–2000. Mortality in both men and women, Panel C: with different calcium concentrations (≥ 2.56, 2.31–2.46, ≤ 2.30 mmol/L). Note: Censored = follow-up period interrupted.

Total mortality was not significantly different between patients with elevated and normal calcium concentrations (see Table III). Men with elevated calcium concentrations had a tendency towards higher mortality compared with normocalcaemic male patients. This was most pronounced between seven and 14 years of follow-up (see Figure 2, panel A, and Table III). There was an increase in cancer mortality (p = 0.039) in men. A comparison with the background population revealed a 2.3 times increased mortality rate (SMR 2.3, 95% CI 1.3–3.8) in men, but not in women with elevated calcium concentrations.

**Table III.** Time to death in years in patients with different causes of death: Comparison between patients with elevated and normal calcium concentrations.

	Total		Men		Women	
	Mean with 95% CI	N/N	Mean with 95% CI	N/N	Mean with 95% CI	N/N
Total mortality						
Elevated Ca^1^	11.4 (10.4–12.5)	60/127	10.3 (8.1–12.5)	14/28	11.7 (10.5–12.8)	46/99
Normal Ca^2^	11.7 (11.0–12.5)	107/254	12.8 (11.3–14.2)	18/56	11.4 (10.5–12.2)	89/198
p-value^3^	0.532		0.109		0.892	
Cancer mortality						
Elevated Ca^1^	14.5 (13.7–15.3)	20/127	12.5 (10.5–14.6)	7/28	14.9 (14.1–15.7)	13/99
Normal Ca^2^	15.0 (14.5–15.5)	28/254	15.4 (14.5–16.2)	5/56	14.8 (14.2–15.4)	23/198
p-value^3^	0.234		0.039*		0.832	
Cardiovascular mortality						
Elevated Ca^1^	12.8 (11.9–13.8)	40/127	11.9 (9.8–14.0)	9/28	13.0 (11.9–14.0)	31/99
Normal Ca^2^	12.4 (11.7–13.1)	88/254	13.1 (11.6–14.5)	16/56	12.1 (11.3–13.0)	72/198
p-value^3^	0.572		0.579		0.338	

Notes: Mean = mean time (years) to death; CI = confidence interval; N = number of events; n = size of group; Ca = calcium.

^1^Patients with elevated calcium concentration.

^2^Patients with normal calcium concentration.

^3^Comparisons between patients with normal and elevated calcium concentration l by Log Rank test.

*Statistically significant difference between the two groups.

The mortality among patients with different concentrations of calcium is shown in Figure 2C. We found a significantly higher mortality (p = 0.004) in patients with low calcium concentrations, but not in high calcium concentrations (p = 0.08), when compared with the middle calcium category of 2.31–2.46 mmol/L. The pattern of mortality diagnoses in patients with low calcium concentrations was the same as that of the background population (data not shown).

## Discussion

This study shows that repeated high calcium concentrations were associated with different diseases and prognoses in men and women, with pHPT being the most common in women. In men, cancer mortality was increased compared with normocalcaemic patients and with total mortality compared with the background population.

The study underscores the importance of further careful investigation of patients with increased calcium levels as at least 88% of these patients will turn out to have an underlying disorder during a 10-year follow-up period. An obvious explanation was found in only 45% of the patients at the initial visit. The delay in diagnosis may be due to vague symptoms, a calcium concentration just above the upper reference interval, or to the physicians being influenced by the debate on the minor significance of slightly deviating calcium concentrations [12,13].

The increase in mortality in patients with elevated calcium concentrations is in line with earlier studies [14]. An increased mortality, specifically in men, was shown in a large population-based study from Sweden, where it was largely attributable to cardiovascular diseases in younger men, although malignant diseases also contributed [15]. While pHPT, which is more common in women with hypercalcaemia, has been associated with increased mortality [16], this could not be demonstrated in this study, which is in line with earlier studies from Tromsö [17]. A possible explanation may be that 43% of patients with pHPT in this study were surgically treated, which may have reduced the mortality as has been shown previously [18], or that the hypercalcaemia was only mild or moderate. It has been pointed out that elevated calcium concentrations are a risk factor for myocardial infarction in middle-aged males [19]. This investigation could not verify this, maybe because the sample size was too small.

This study showed that the calcium concentration had an impact on total mortality. Low calcium concentrations were associated with higher mortality. Several studies from intensive care units have shown that a low calcium concentration is associated with increased mortality without any specific underlying cause [20–22]. In primary care, a low calcium concentration may be due to vitamin D deficiency, malnutrition, kidney and intestinal disease, or a malignancy-dependent low albumin concentration.

One limitation of this study was that the inclusion of the calcium analysis for this patient group was based on a single calcium concentration measurement, entailing a risk of including erroneously transient high calcium concentrations during one period. This highlights the importance of rechecking any spurious laboratory test [23]. For this reason we have chosen the term elevated calcium concentration instead of hypercalcaemia. Another limitation was that the controls were also patients, with various disorders, at the HCC. An age- and sex-matched random sample from the population would have been the best choice, but since this was a retrospective study from the start it was not possible. For this reason, the mortality analysis was also performed against the background population. Another limitation is the small study sample, as the study was performed in a single HCC; hence the results should be interpreted with caution, especially for the subgroups.

The strength of this study was the high participation rate of 86% at follow-up, probably primarily due to the study being limited to one HCC. Another advantage is that this study mirrors the common general practice consultation.

In conclusion, a single elevated calcium concentration is not reliable and should be investigated further. One decade after the detection of a single elevated calcium concentration, the number of patients without an underlying disorder fell from 55% to 12%. Elevated calcium concentrations were associated with pHPT in women, and with increased cancer mortality in men. The clinical consequence of persistent hypercalcaemia in men should be to consider further examinations to detect cancer or possible cardiovascular disease. Women with persistent hypercalcaemia should be investigated for suspected pHPT, but cancer cannot be ruled out. It is important to recheck elevated calcium levels, as the results may fluctuate.
